# Current Treatment Options in Homozygous Familial Hypercholesterolemia

**DOI:** 10.3390/ph16010064

**Published:** 2022-12-31

**Authors:** Meral Kayikcioglu, Lale Tokgozoglu

**Affiliations:** 1 Department of Cardiology, Medical Faculty, Ege University, 35100 Izmir, Turkey; 2 Department of Cardiology, Medical Faculty, Hacettepe University, 06230 Ankara, Turkey

**Keywords:** familial hypercholesterolemia, guidelines, lipid lowering therapy, low-density lipoprotein cholesterol, apheresis, Lomitapide, Evinacumab

## Abstract

Homozygous familial hypercholesterolemia (HoFH) is the rare form of familial hypercholesterolemia causing extremely high low-density lipoprotein cholesterol (LDL-C) levels, leading to atherosclerotic cardiovascular disease (ASCVD) in the first decades of life, if left untreated. Early diagnosis and effective lipid lowering therapy (LLT) are crucial for the prevention of early ASCVD in patients with HoFH. On-treatment LDL-C levels are the best predictor of survival. However, due to the absent or defective LDL-receptor activity, most individuals with HoFH are resistant to conventional LLT, that leads to LDL-C clearance by upregulating LDL-receptors. We are at the dawn of a new era of effective pharmacotherapies for HoFH patients, with new agents providing an LDL-receptor independent cholesterol reduction. In this context, the present review provides a summary of the currently available therapies and emerging therapeutic agents for the management of patients with HoFH, in light of recent evidence and guideline recommendations.

## 1. Introduction

Homozygous familial hypercholesterolemia (HoFH) is the severe rare form of familial hypercholesterolemia (FH), causing extremely high low-density lipoprotein (LDL)-cholesterol (LDL-C) levels, leading to atherosclerotic cardiovascular disease (ASCVD) in the first decades of life, if left untreated [1,2,3]. The estimated prevalence of HoFH ranges between 1 in 300,000–1 in 360,000. The prevalence is higher in isolated populations and in populations with the founder effect [2]. 

There are at least four genes including *LDLR*, *APOB*, *PCSK9*, and *LDLRAP1*, that cause HoFH, with phenotypic variation [3,4]. In most of the HoFH cases (>90%), biallelic mutations are in the *LDLR* gene leading to significant changes in LDL-receptor activity, associated with typically LDL-C levels exceeding 400 mg/dL (>10 mmol/L) [2,3,4]. Patients with biallelic mutations may be either ‘*simple homozygous*’ with two identical copies of the same allele or ‘*compound heterozygous*’ with nonidentical alleles. Another type of HoFH is ‘*double heterozygosity’*, with two heterozygous mutations in two different genes regulating cholesterol levels. By altering LDL-receptor activity and reducing LDL-C uptake from circulation, these genetic changes result in a marked increase in the circulating LDL-C levels starting at birth.

Early diagnosis and effective lipid lowering therapy (LLT) are crucial for the prevention of early ASCVD in patients with HoFH. On-treatment LDL-C levels are shown to be the best predictor of survival and the sooner the onset of the treatment, the lower the cardiovascular consequences [5]. However, due to absent or defective LDL-receptor activity, most individuals with HoFH are resistant to conventional therapies that lead to LDL-C clearance by upregulating LDL-receptors. Response to conventional LLT is determined by the residual LDL-receptor activity. Patients with < 2% of LDL-receptor activity, i.e., those with null variants of *LDLR (simple homozygous or compound heterozygous)*, cannot respond to conventional LLT including statins, ezetimibe, and PCSK9 inhibitors. On the other hand, patients with defective mutations in *LDLR* may have some degree of response to these agents [2]. Therefore, despite similar phenotypes, patients may have completely different responses to LLT. Even if there is LDL-receptor activity, the LDL-C reduction afforded by these therapies are not adequate to get to LDL goals, as these patients require immense reductions in LDL-C levels [6]. Therefore, clinical management of these patients is significantly challenging, and LDL-apheresis is the most effective means of treatment in these patients [7]. However, the expensive, semi-invasive, and time-consuming nature of apheresis causes decreased quality of life, increased risk of depression, and deteriorations in mental status [8,9], leading to high refusal and low adherence.

We are at the dawn of a new era of effective pharmacotherapies for HoFH patients, with new agents providing LDL-receptor independent cholesterol reduction. The purpose of this review is to provide a summary of the currently available therapies and emerging therapeutic agents for the management of patients with HoFH, in light of recent evidence and guideline recommendations.

## 2. Treatment of Patients with HoFH

The treatment algorithm and its goals in patients with HoFH should be handled like other patients with high or very high risk [10]. Lifestyle changes and combatting other risk factors should be started as soon as these patients are diagnosed [3]. All patients with HoFH, should be advised and encouraged to consume a heart-healthy diet (low in saturated fat) and to participate in appropriate levels of physical activity [3,11]. The mainstay of the treatment of these patients is to adopt an aggressive pharmacotherapy strategy with multiple therapeutics, enabling LDL-C goals of < 55 mg/dL (1.4 mmol/L) for those with very high risk, and 70 mg/dL (1.8 mmol/L) for those with high risk, as early as possible [12]. As most of these patients are diagnosed in childhood or infancy, implementation of such an aggressive treatment is challenging. Therefore, patients should be followed in experienced centers and close collaboration with their families should be established [2]. Current guidelines recommend an LDL-C target of < 135 mg/dL (3.5 mmol/L) for children [12].

**Figure 1:**
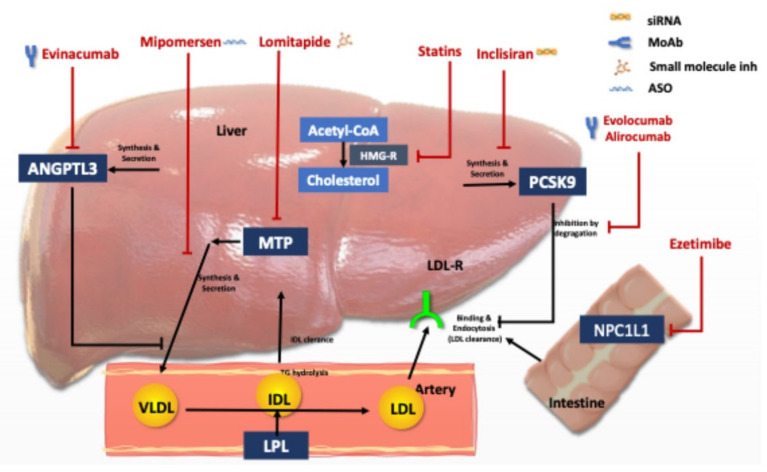
Lipid lowering agents used for Homozygous FH treatment and their mode of action. Abbreviations: LDL: Low density lipoprotein, LDL-R, LDL receptor; PCSK9, proprotein convertase subtilisin/kexin type 9, HMG, 3-hydroxy-3-methylglutaryl, MTP, microsomal triglyceride transfer protein; VLDL, very-low-density lipoprotein, IDL; intermediary density lipoprotein, ANGPT3: angiopoietin-like protein family 3, and NPC1L1, Niemann-Pick C1-like protein 1.

Table 1 and Figure 1 depict the currently available options of LLT for HoFH, and Table S1 (Supplementary) summarizes the pending and conducted clinical studies of LLT in these patients. The main determinant in achieving the treatment goals in HoFH is the underlying genetic mutation, i.e., the residual LDL-receptor activity. Patients with HoFH show a broad spectrum of variable responses to conventional and emerging LLT [13] due to genetic and phenotypic variants. However, routine testing of genetics or LDL-receptor activity is not recommended for guiding the therapeutic approach [12]. The treatment should start with conventional statin therapy, and depending on the response, combination therapy should be added and/or non-pharmacologic interventions considered.

## 3. Conventional Lipid Lowering Therapy

Statins and ezetimibe are still the first line therapy for patients with HoFH, although their mechanism of action is LDL-receptor dependent. Long term studies looking at survival of HoFH patients in the post-statin era have shown their prognosis to be significantly better compared to the pre-statin era [14]. Statins alone or in-combination with ezetimibe have been shown to decrease ASCVD mortality in both adult and pediatric HoFH patients [14,15]. Response to statins and ezetimibe could be more than expected in double heterozygous patients, and especially those with sitosterolemia who have a greater response to ezetimibe [3,7,16]. The use of resins (bile acid sequestrates) is limited mostly due to their poor tolerance and low efficacy; they are used mainly in pediatric patients and pregnant women with FH, with their advantage of not being absorbed [2,3]. However, the effect of all these conventional agents is not sufficient to achieve adequate LDL-C reduction, and more effective add-on LLTs are required in most cases. Moreover, a recent meta-analysis of 94 studies conducted on HoFH patients showed that the age-onset of major adverse cardiovascular events has been delayed 11 years in the post-1990 compared to the pre-1990 era, but the prevalence of ASCVD has not changed, emphasizing the need for new treatment approaches on top of conventional LLT [17].

## 4. PCSK9 Inhibitors

Proprotein convertase subtilisin/kexin type 9 (PCSK9) is an important regulator of cholesterol metabolism. PCSK9 is secreted from the hepatocytes to the plasma, where it binds to the LDL-receptors, facilitating the lysosomal degradation of these receptors. Thus, inhibition of PCSK9, increases the expression of LDL-receptors on the cell membrane, thereby increasing the clearance of LDL-C. Inhibition of PCSK9, either by monoclonal antibodies or RNA-based therapeutics have become a popular area of research during the last 15 years, as a promising drug target, especially for patients with FH or resistant hypercholesterolemia [2,3].

### 4.1. Alirocumab and Evolocumab

Alirocumab and evolocumab are fully humanized monoclonal antibodies that enable most of the patients with FH to reach their LDL-C goals. These agents require monthly doses or dosing every 2-week. Both are now recommended as the third-line treatment for patients with FH receiving maximally tolerated LLT, based on the RCTs and long-term real-life data [18]. The efficacy of these monoclonal inhibitors of PCSK9 in HoFH patients depends on the LDL-receptor activity [19]. Based on the findings of the ‘Inhibition of PCSK9 with evolocumab in homozygous familial hypercholesterolaemia (TESLA-B) trial conducted on HoFH patients, the use of 420 mg evolocumab, once monthly for 3 months, is associated with a 31% LDL-C reduction for HoFH patients with at least 2% of functioning LDL-receptors, who were already on stable treatment that did not include apheresis [20]. The open-label long-term outcomes study of evolocumab, at a dose of 420 mg, administered once monthly or every 2 weeks, confirmed an almost 25% additional LDL-C decrease in HoFH patients receiving LDL apheresis [21]. Similarly, ODYSSEY HoFH trial resulted in a 35% decrease in LDL-C levels with 12-week treatment of alirocumab, at a dose of 150 mg, administered every 2 weeks, in 69 patients with HoFH, who were on stable conventional LLT (statins and ezetimibe), with a baseline LDL-C level of 295 mg/dL (7.6 mmol/L) [22]. The additional LDL-C reduction was 17.9% in the 6 HoFH patients on stable apheresis, in the ODYSSEY HoFH Trial. The LDL-C reduction with PCSK9 inhibitors might be variable, ranging from 7% to 56%, in receptor defective patients, even with the same mutation [20]. Therefore, unless patients are known to be receptor negative, a therapeutic trial is recommended with these agents, if treatment goals cannot be attained [23]. Patients with a response of 10–15% LDL-C reduction should continue PCSK9 inhibitors, as the range of 10–15% is important and likely to be associated with an incremental decrease in ASCVD events [23]. However, a 10–15% decrease in LDL-C is far from the LDL goals and additional therapeutics are needed. Evolocumab has been approved for HoFH treatment in adults and children > 12 years of age. PCSK9 inhibitors should be injected subcutaneously after the apheresis procedures, in HoFH patients on regular apheresis. Both evolocumab and alirocumab have been shown to be safe and well tolerated. The most common side effect is injection site reaction. The recent Fourier-OLE (open label extension) showed that the safety of evolocumab on long-term (>8 years) use was similar to that observed in the original placebo arm of the Fourier trial [24].

### 4.2. Inclisiran

Inclisiran is a small interfering ribonucleic acid (siRNA) that inhibits the intracellular synthesis of PCSK9 in hepatocytes, thereby upregulating the LDL-receptors. The initial dose is 300-mg delivered subcutaneously, and the next dose is injected at 3 months, followed by dosing every 6-month thereafter. The infrequent dosing regimen will have advantages especially in young individuals with FH and may result in improved compliance and comfort. Moreover, the infrequent dosing regimen can provide an advantage during the outbreaks and lockdown time for subjects with HoFH [25]. Inclisiran received approval in December 2020 in the EU and in December 2021 by FDA for adults with primary hypercholesterolemia (HeFH or non-familial) with the results of a series of randomized controlled trials (RCTs), the ORION trial program.

In phase II and III Orion studies, Inclisiran has shown to lower LDL-C up to 50% in a broad range of patients with hypercholesterolemia, including those with HeFH and HoFH [26]. The incidence of adverse events did not differ between the groups [27]. Similar to monoclonal PCSK9 inhibitors, the response to inclisiran in patients with HoFH is lower than that in those with HeFH, and is variable. In the pilot study of 4 patients with HoFH (ORION 2), inclisiran showed an LDL-C reduction ranging from 17.5% to 37.0% at day 180 in 3 patients, but the fourth patient was not responsive [2,27]. The ongoing ORION 5 study which is a long term RCT (NCT03851705) will address the efficacy and safety of inclisiran in HoFH patients, with or without lipid apheresis. Similar to the monoclonal PCSK9 inhibitors, inclisiran has a favorable safety and tolerability profile [26,27].

### 4.3. Lerodalcibep

Lerodalcibep (formerly LIB 003), is a small recombinant fusion protein of a PCSK9 binding domain (adnectin) and albumin, which has demonstrated a highly effective PCSK9 and LDL-C suppression, with monthly (QM) 300 mg subcutaneous dosing in Phase 2 studies. Phase 3 RCT (OLE trial-NCT04034485), comparing the safety and efficacy of *Lerodalcibep* to evolocumab in HoFH patients, on stable oral LLT not receiving LDL-apheresis, has completed recruitment.

## 5. Pharmacologic Agents Acting Independent of LDL-Receptors

### 5.1. Anti-Apo-B Therapies

#### 5.1.1. Lomitapide

Lomitapide is a new generation potent lipid lowering agent with a mechanism of action independent of LDL-receptors, approved for the treatment of HoFH. Lomitapide inhibits microsomal triglyceride-transfer protein (MTP), a cellular protein responsible for the transport of neutral lipids between membrane vesicles, acting as a chaperone for the synthesis of ApoB-containing triglyceride-rich lipoproteins. MTP has a critical role in the assembly and secretion of ApoB-containing lipoproteins in the liver and intestines [28]. Therefore lomitapide, besides triglycerides, effectively reduces LDL-C levels in patients who are lacking or have defective LDL-receptors. Lomitapide has been approved by the FDA as a lipid-lowering agent for patients with HoFH as an adjunct to standard LLT, and by the European Medicines Agency (EMA), for those treated with standard LLT with or without apheresis.

The phase 3 open-label dose-escalation trial demonstrated that lomitapide at maximal-tolerated doses (5–60 mg/day) reduces LDL-C by 50% at 26 weeks, added to standard of care, including lipid apheresis in 29 HoFH patients [29]. A phase 3 extension study which was conducted in Japanese patients with HoFH (*n* = 8) also showed 50% LDL-C reduction at 60 weeks, with no additional safety concerns [30]. Long term lomitapide treatment (range 2.1–5.7 years) with a median dose of 40 mg/day reduced LDL-C levels on an average of 45.5%, in 19 HoFH adults [31]. Of note, during the 246-week therapy, 74% (*n* = 14) patients achieved the LDL-C target of 100 mg/dL and 58% (*n* = 11) patients target of 70 mg/dL on at least 1 occasion. The efficacy of lomitapide is also comparable to lipid apheresis. The long-term LDL-C lowering effectiveness of these two treatments were evaluated by comparison of two independent HoFH cohorts treated with lomitapide (Italy) or Apheresis (France) [32]. The percentage of subjects that achieved a yearly on-treatment LDL-C percent reduction of > 50% from baseline was almost 3 times higher in the Lomitapide cohort than in the apheresis cohort (77.3% vs. 24.1% *p* < 0.001). In our experience, even low doses of lomitapide could reduce the frequency of lipid apheresis [33]. There are also cases in the literature with cessation of apheresis therapy with this agent [13]. Up to 40 to 80% apheresis cessation rates are reported from different registries [13,33]. Therefore, in current practice lomitapide is offered to HoFH patients who have failed to reach treatment targets while on a combined therapy of apheresis and conventional LLT, and have had a trial of evolocumab [23]. However, with availability of lomitapide and similar novel effective agents, these agents (lomitapide and evinacumab) are becoming second line therapy for HoFH, before the initiation of therapeutic apheresis.

Hepato-steatosis is a well-known adverse effect associated with lomitapide therapy. But this is a unique consequence of all Apo-B targeting therapies. Other commonly reported side effects include increases in transaminase levels, patient tolerability, and gastrointestinal side effects, such as nausea and diarrhea, which have been shown to diminish in severity and incidence over time, with the use of lomitapide [31]. Tolerability can be easily improved with an effective low fat diet modification, but patients should be educated on how to improve adherence. Long-term follow-up assessing safety, up to a median 5.1 years, in patients who participated in the phase 3 study did not show any new safety concerns [31]. Moreover lowering the dose of lomitapide (10–20 mg/dL) as assessed by the LOWER study, has resulted in lower adverse event rates in a 5-year follow-up, compared with the results of the Phase-3 study conducted with a dosing of 40–60 mg/dL [34]. Furthermore, the addition of lomitapide at an average dose of only 19 mg/day to background LLT has led to a mean 68.2% reduction in LDL-C levels in 15 patients with HoFH [35]. At their last visit, 60% of patients showed LDL-C < 100 mg/dL (2.6 mmol/L) and 46.6% < 70 mg/dL (2.8 mmol/L). More importantly, 80% (8 of 10 patients) given apheresis were able to stop apheresis completely [35]. Similar results were also observed in the pan-European Registry [36]. Of note, pan-European Registry was a multicenter retrospective observational study, which included 75 HoFH patients treated with lomitapide, reflecting the real-world clinical settings from 9 European countries, with an almost 9-year follow-up.

Lomitapide has also been shown to have good safety, tolerability, and effectiveness in the pediatric population. In a recent study it was safely used in children as young as 4 years of age [37]. A clinical trial of lomitapide in pediatric HoFH patients is currently recruiting (NCT04681170).

#### 5.1.2. Mipomersen

Mipomersen is a second-generation antisense oligonucleotide (ASO) inhibitor targeting ApoB. It is delivered by weekly subcutaneous injections. Although it is not approved in Europe, it is approved and available in the USA for HoFH patients not on apheresis. In a prospective phase II single center RCT of 15 patients, mipomersen has decreased the pre-apheresis LDL-cholesterol and Lpa levels significantly, mean-while seven patients have discontinued the drug due to side effects.

### 5.2. ANGPTL3 Inhibitors

Three members of the angiopoietin-like (ANGPTL) protein family-ANGPTL3, ANGPTL4, and ANGPTL8 are significant regulators of plasma lipoproteins [38]. They inhibit lipoprotein lipase (LPL), which plays a key role in intravascular lipolysis of triglycerides. ANGPTL3 emerged as a new target from genetic studies. Both loss of function(LOF) variants and inactivation of the *Angptl3* gene are associated with significantly reduced levels of triglycerides and LDL-cholesterol. LOF mutations also have been shown to be associated with markedly reduced risk of developing ASCVD [39]. These findings accelerated the LLT development, targeting the inactivation of ANGPTL3, using either a specific monoclonal antibody, ASOs, and/or siRNAs.

#### 5.2.1. Evinacumab

Evinacumab is a monoclonal antibody that inhibits ANGPTL3, thereby significantly reducing both LDL-C and triglycerides. It was recently approved in Europe and the US for its use in patients with HoFH. It is administered with intravenous infusion, at a dose of 15 mg/kg of body weight monthly. In a phase 3 RCT of patients with HoFH (*n* = 65) (ECLIPSE study), evinacumab therapy provided a 47% LDL-C reduction at 24 weeks, regardless of being treated with or without apheresis [40]. Of note, the reduction was similar in those with and without the *LDLR* null variants, denoting the LDL-receptor independent mechanism of action. With a favorable safety profile, good tolerability, and a remarkable LDL-C lowering response, evinacumab seems to have the potential to ease the hurdles in the management of HoFH [2,3].

#### 5.2.2. RNA Based Treatments Targeting ANGPTL3

Several RNA-targeted therapeutics to inhibit ANGPTL3 are being tested. ARO-ANG3, a siRNA targeting the *ANGPTL3* gene is currently being tested in a phase 1 clinical trial, with 40 healthy volunteers and over 50 subjects with dyslipidemia (NCT03747224). Initial results of the healthy volunteers showed that subcutaneously delivered ARO-ANG3 on day 1 and 29 leads to a 45–54% reduction in LDL-C levels at 4–6 weeks, following the second injection [41]. ARO-ANG3 seems to provide less frequent dosing like inclisiran [2].

The first results of an ASO against ANGPTL3 in patients with hypertriglyceridemia and FH also have been recently reported [42]. In a mice model of null variant of HoFH, co-treatment with ASOs against *MTP* and *ANGPTL3* is tested. Such a combination therapy might be promising in alleviating the hepatic lipid accumulation due to MTP inhibition.

## 6. Interventions to Lower LDL Independent of LDL-Receptor

### 6.1. Lipoprotein Apheresis

**Figure 2:**
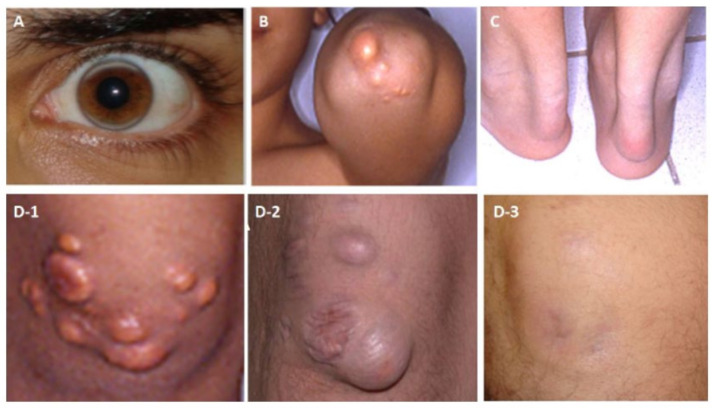
Examples for cholesterol depositions of a patient with HoFH (**A**) Arcus lipemia (at age 23 years, while on a regular apheresis treatment for 3 years), (**B**) Cholesterol deposition on the extensor surface of the elbow (at age 13), (**C**) Thickening of the Achilles tendon due to cholesterol deposition (at 13 years of age) (**D1–3**) Deposition in the skin on the extensor surface of the knee-joint (**D-1**) Before apheresis at 13 years of age, (**D-2**) After a 3-year discontinuation of apheresis at 21 years of age, and (**D-3**) Cholesterol deposition on the extensor surface of the knee-joint disappearing after 3 years of regular apheresis.

Lipoprotein apheresis, which has been in use for more than 45 years, is the most effective means of lowering LDL-C levels in patients with HoFH [7,33,43]. There are several lipid apheresis methods, but in general all are almost equal in selectively removing the circulating Apo-B containing atherogenic lipoproteins, including lipoprotein (a) [33,43]. Apheresis also reduces inflammatory markers, oxidative stress, and thrombogenic factors, and improves endothelial functions. Regular apheresis treatment can effectively and safely induce the regression of xanthomas (Figure 2), retards the progression of atherosclerotic lesions, and improves survival [1,7,33,43]. Depending on the amount of volume filtered, apheresis eliminates approximately 60% of LDL-C from the circulation, per session. However, apheresis is an intermittent therapy that leads to a saw-tooth like pattern of LDL-C levels [23,33]. Therefore, the extent of the rebound of the LDL-C levels may diminish the expected benefits of apheresis therapy. This rebound increase of LDL-C should be alleviated by increasing the frequency of the procedures and with concomitant use of LLT [8,23,33,43]. Thus, apheresis should be performed frequently, i.e., the ideal is on a weekly manner.

The initiation age of apheresis is associated with the incidence of cardiovascular events during regular apheresis therapy. Indeed, LDL-apheresis should be initiated before the age of 6–7 years, to prevent the progression of aortic root atheroma [33,43]. However, in clinical practice, most patients experience ineffective apheresis, and fail to reach LDL-C targets even in countries where LDL apheresis is widely available [7,8,9,33]. This real-world care failure is due to several factors including late diagnosis, delayed referral to apheresis, and improper frequency of apheresis procedures. All these denote that awareness is still low among physicians caring for HoFH patients [44]. Moreover, the semi-invasive, time consuming, and the chronic nature of lipid apheresis significantly contributes to the high refusal and low adherence rates [7,8,9,33]. Hence, a structured approach, including standardized protocols for apheresis therapy, with regular cardiovascular follow-up by an experienced multidisciplinary team, is warranted [10,33]. Another important aspect of the management of HoFH patients on apheresis therapy is the need for a close follow up of LDL-C levels with time-averaged LDL-C. Of note, the Kroon formula calculates the time-averaged LDL-C between two apheresis procedures, adjusting for the non-linear rebound of LDL-C. The formula is as follows: LDL_mean_ = LDL_min_ × *K* (LDL_max_ − LDL_min_), where LDL_min_ = LDL-cholesterol immediately after apheresis and LDL_max_ = LDL-cholesterol immediately prior to apheresis (*K* coefficient is 0.73 for HeFH and 0.66 for HoFH) [23,33,45]. If the target time averaged LDL-C levels cannot be attained, new effective lipid lowering agents with documented cardiovascular benefit, should be added immediately to the treatment algorithms of HoFH patients on apheresis therapy [33]. HICC registry documented that getting to goals is almost possible with 4 to 5 therapeutics including LDL-apheresis [44]. Moreover, such effective/potent combinations may enable a decrease in the frequency of apheresis procedures or even cessation of apheresis treatment, as reported with lomitapide use in real world experiences [33,34,35,36].

### 6.2. Liver Transplantation

Liver transplantation is considered as a curative treatment or last resort of HoFH, with substantial LDL-C reductions of up to 80% (47). Like apheresis, it has been shown that coronary artery disease and cholesterol stigmata regress: however, if triggered, the progression of aortic stenosis cannot be prevented after liver transplantation [33,46,47]. Liver transplant has noticeable drawbacks due to the scarcity of donors, high risk of surgical complications, on top of the life-long requirement of immunosuppressive therapy, for the prevention of graft rejection [46,47,48]. Moreover, the existing evidence may harbor severe bias as the available follow-up is for short term, in almost all the presented case and case series. A recent case series of 9 patients with HoFH with up to 28 years (mean 11.7 ± 11.7 years) follow-up after therapeutic liver transplantation, drew attention to the inherent mortality risk and severe complications of this treatment modality [48]. Moreover, achieving the LDL-C targets after liver transplantation is not conclusive, as patients may still require aggressive LLTs. These drawbacks limit the use of liver transplant as a curative treatment of HoFH.

## 7. Future or near Future Aspects

The great progression of biotechnologies enabled the rapid development of new therapeutic options, which are promising in gaining the LDL-C targets in individuals with HoFH. Besides the mentioned mAb and RNA-based therapeutics, gene transfer, silencing, and editing are currently under investigation for HoFH [2,3].

### 7.1. CRISPR-Based Genome Editing

Clustered regularly interspaced short palindromic repeats (CRISPR)-Cas-mediated genome editing has also been successfully used to lower LDL-C levels, by targeting *ANGPTL3* in *LDR* knocked out mice, where adenoviral vectors were used to deliver the CRISPR base editors [49]. However, lipid nanoparticles may be less immunogenic than a viral vector [50]. Animal models using lipid nanoparticles to deliver CRISPR base editors modifying *PCSK9* have been completed successfully, with a 60% reduction in LDL-C levels [51].

### 7.2. Gene Transfer

With the discovery of the adeno-associated virus as a vector, the delivery of the transgene became more feasible and safer, especially in the treatment of monogenic diseases [52]. Of note, adeno-associated virus vector is less immunogenic and, more importantly, poses a lower risk of integration into the host genome, compared with other viral vectors. However, transaminase elevations have been reported with adenoviral mediated gene transfer, due to a T-cell immune response against the vector’s capsid [52,53], which is alleviated with steroid pre-treatment.

The preclinical research using the adenoviral mediated gene transfer in *LDLR* knocked out mice, has resulted in successful expression of LDL-receptors in the liver, with a significant decrease in LDL-C levels and regression of atherosclerosis, with no significant adverse events [54,55,56]. An early phase first-in-human study (NCT 02651675) of an adeno viral mediated *hLDLR* gene transfer was recently completed in HoFH patients (*n* = 9), results are pending.

## 8. Conclusions

**Figure 3:**
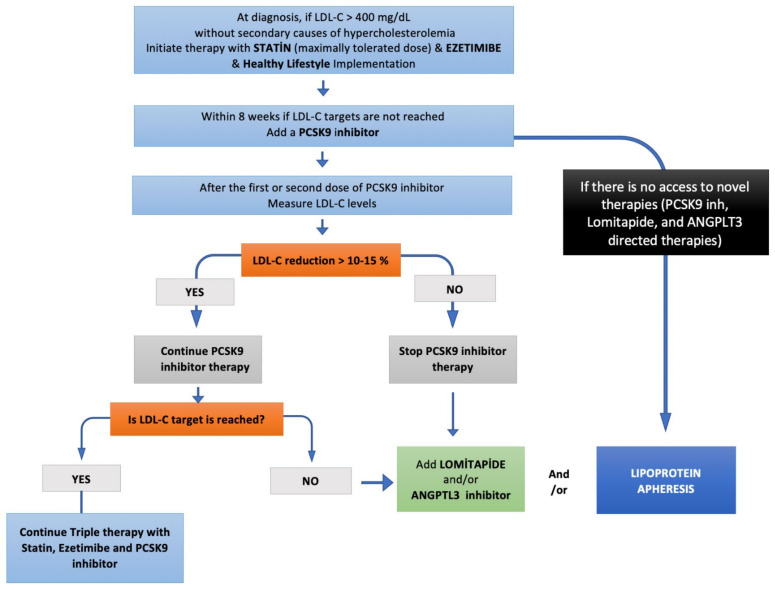
Step-by-step HoFH treatment algorithm.

The mortality and morbidity are mainly determined by on-treatment LDL-C levels in patients with HoFH. Conventional LLT including statins, ezetimibe, and PCSK9-inhibitors constitute the first-line therapy of HoFH patients (Figure 3). As all these 3 agents act via LDL-C clearance, by upregulating LDL-receptors, most of the HoFH patients, especially those with null/null *LDLR* mutations, do not reach LDL-C targets. Therefore, most HoFH patients are referred to LDL-apheresis as the most effective means of LDL-C lowering. However, LDL-apheresis is an expensive, semi-invasive, and time-consuming therapy that is associated with decreased quality of life and impaired mental status, leading to high refusal and low adherence rates. Lomitapide and evinacumab are more effective pharmacotherapies for HoFH patients, providing an LDL-receptor independent cholesterol reduction. Recent evidence denotes the importance of the combination of conventional LLT and newer LDL-receptor independent therapies to get the LDL-C targets. Therefore, HoHF patients should be adopted an aggressive pharmacotherapy strategy with multiple therapeutics, enabling LDL-C goals of < 55 mg/dL (1.4 mmol/L) for those with very high risk, and 70 mg/dL (1.8 mmol/L) for those with high risk as early as possible.

## Figures and Tables

**Table 1:** Lipid lowering therapies for patients with Homozygous familial hypercholesterolemia.

Agent	Mechanism of Action	Route & Dose	Effect on Lipoprotein Levels in HoFH	Clinical Study Results	Guideline Recommendations	Comment
LDL-R dependent						
**Statins**	-HMG-CoA reductase inhibition, -Reduce cholesterol synthesis, -Upregulate LDL-R -Increase LDL-C clearance	PO, Dose depends on the LDL-C target & type of statin	LDL-C 14–31%↓	Lower both LDL-C & ASCVD risk in all statin trials in both primary & secondary prevention.	First line in HoFH with limited efficacy	- Cornerstone of LDL-C-lowering therapy in FH -More effective in ARH patients
**Ezetimibe**	-Inhibits NPCILI protein -Inhibits cholesterol absorption -Upregulate LDL-R -Increase LDL-C clearance	PO, 10 mg/day	LDL-C 5–14%↓	Lowers both LDL-C & ASCVD outcomes on top of statins	Second line add on therapy for HoFH	-Approved for treatment of HeFH & HoFH patients either alone or in combination with statins -More effective in sitosterolemia
**Bile acid sequestrants** **(Cholestyramine, Colestipol & Colesevelam)**	-Decrease reabsorption of bile acids -Reduce cholesterol content in hepatocytes -Upregulate LDL-R -Increase LDL-C clearance	PO, Daily	LDL-C 0–10%↓	No RCT on FH	None for FH	-Mostly preferred in children and pregnant women -Effect is weak. -Not absorbed
**PCSK9 inhibitors (Alirocumab, Evolocumab)**	-Monoclonal antibodies to PCSK9 -Inhibits PCSK9, -Upregulate LDL-R -Increase LDL-C clearance	Alirocumab SC, Biweekly 75–150 mg Evalocumab SC, Biweekly 140 mg, Monthly 420 mg	Alirocumab HoFH LDL-C 26%↓ Evalocumab HoFH LDL-C 15–32%↓	-Reduced LDL-C & ASCVD outcomes in phase 3 studies. Alirocumab -ODYSSEY-HoFH Evalocumab -TESLA -HoFH short term -TAUSSING-HoFH&HeFH 4years sustained efficacy & safety	Treatment with a PCSK9 inhibitor is recommended in very-high-risk FH patients if the treatment goal is not achieved on maximal tolerated statin plus ezetimibe.	- Both are beneficial in HoFH patients with at least 2% of functional LDL-Rs.
**Inclisirian**	-SiRNA inhibiting the translation of PCSK9 -Upregulate LDL-R -Increase LDL-C clearance	SC, 300 mg on Days 1, 90 then every 6 months	HoFH LDL-C 12–37%↓	-Orion Trial Program -Orion-5 Phase 3 RCT on HoFH-ongoing	-Not in the GLs yet -Approved for adults with primary hypercholesterolemia or mixed dyslipidemia by EC December 2020	-Enables infrequent dosing & sustained effect -Promising use in young FH individuals with an improved compliance
**LDL-R independent action**						
**Lomitapide**	-Inhibits MTP, thereby interfering with the assembly of lipoproteins -Decrease ApoB	PO, 10–60 mg/day	LDL-C 24–52%↓	-Effective in phase 3 -LOWER registry (5-year real life data) showed efficacy even in low doses (10–40 mg/d)	-Approved by EMA &FDA as an adjunct to LLT in patients with HoFH ≥ 18 years with/without apheresis	-Shown to either reduce the frequency of apheresis or replace apheresis
**Evinacumab**	-Monoclonal antibody to ANGPTL3	IV inf, monthly	LDL-C 47.1%↓	-ECLIPSE study showed efficacy & safety in HoFH -ASCVD study not done yet.	-Not in the GLs yet - Approved by FDA for HoFH adults & children aged ≥ 12 years in December 2020	-Effective also in Null variants -Advantage of monthly injections -Effective in lowering TGs

ACL: Adenosine triphosphate-citrate lyase; AMPK: Adenosine monophosphate-activated protein, kinase; ANGPTL3, angiopoietin-like protein 3; Apo: Apolipoprotein; ASCVD, atheroscleroticcardiovascular disease FH, familial hypercholesterolemia, HeFH, heterozygous FH; HoFH, homozygous FH; LDL: low-density lipoprotein, LDLR; low-density lipoprotein receptor, MTP = microsomal triglyceride transfer protein; NPC1L1 = Niemann-Pick C1-like 1 protein PCSK9; pro-protein convertase subtilisin kexin 9, pts: patients SC = subcutaneous TG, triglyceride, and VLDL; very-low-density lipoprotein.

## Data Availability

Data sharing not applicable.

## Supplementary Materials

The following supporting information can be downloaded at: https://www.mdpi.com/article/10.3390/ph16010064/s1, Table S1: Summary of pending and conducted clinical studies of lipid lowering agents in HoFH.
