# Supplementary material for: Current Treatment Options in Homozygous Familial Hypercholesterolemia
Source: Pharmaceuticals (Basel). 2022 Dec 31;16(1):64. doi: 10.3390/ph16010064 (PMC9863418; doi:10.3390/ph16010064)
Supplement: Supplementary file 1 [file pharmaceuticals-16-00064-s001.zip › pharmaceuticals-2087609-supplementary.pdf]

**Supp Table S1.** Summary of pending and conducted clinical studies of lipid lowering agents in HoFH (Data is retrived from clinicaltrials.gov – Access date: Dec 2022)							
**NCT Number (Study Acronym)**	**Drug**	**Status**	**Phases<br>Study type**	**Age**	**Conditions (enrollment ;n)**	**Outcome Measures**	**URL**
NCT05611528	Evinacumab	Not yet recruiting	Phase 4<br>Interventional<br>Single Group<br>Open Label	>18 Years	HoFH (10)	Efficacy, Safety, Tolerability	https://ClinicalTrials.gov/show/NCT05611528
NCT04233918	Evinacumab	Active, not recruiting	Phase 3<br>Interventional<br>Single group<br>Open label	5 -11 Years	HoFH (20)	Efficacy, Safety, Tolerability	https://ClinicalTrials.gov/show/NCT04233918
NCT03409744	Evinacumab	Active, not recruiting	Phase 3<br>Interventional<br>Single group<br>Open label	> 12 Years	HoFH (116)	Long term Efficacy, Safety, Tolerability	https://ClinicalTrials.gov/show/NCT03409744
NCT03399786	Evinacumab	Completed	Phase 3<br>Interventional<br>Randomized<br>MaskingQuadruple	> 12 Years	HoFH (65)	Efficacy, Safety, Tolerability	https://ClinicalTrials.gov/show/NCT03399786
NCT02265952	Evinacumab	Completed	Phase 2<br>Interventional<br>Single group<br>Open label	> 18 Years	HoFH (9)	Efficacy, Safety, Tolerability	https://ClinicalTrials.gov/show/NCT02265952
NCT05217667 (GATEWAY)	ARO-ANG 3 Injection	Recruiting	Phase 2<br>Interventional<br>Randomized<br>Open label	> 16 Years	HoFH (16)	Efficacy, Safety, Tolerability	https://ClinicalTrials.gov/show/NCT05217667
NCT03851705 (ORION-5)	Inclisiran	Completed	Phase 3<br>Interventional<br>Randomized<br>MaskingDouble	18 - 80 Years	HoFH (56)	Efficacy, Safety, Tolerability	https://ClinicalTrials.gov/show/NCT03851705

NCT04659863 (ORION-13)	Inclisiran	Recruiting	Phase 3 Interventional Randomized Masking Double	12 - 17 Years	HoFH (12)	Efficacy, Safety, Tolerability	https://ClinicalTrials.gov/show/NCT04659863
NCT03814187 (ORION-8)	Inclisiran	Active, not recruiting	Phase 3 Interventional Single group Open label	> 18 Years	ASCVD, HeFH,|HoFH All (3275)	Efficacy, Safety, Tolerability	https://ClinicalTrials.gov/show/NCT03814187
NCT02963311 (ORION-2)	Inclisiran	Completed	Phase 2 Interventional Single group Open label	> 12 Years	HoFH (9)	Efficacy	https://ClinicalTrials.gov/show/NCT02963311
NCT01412034 (MODE)	CER-001 (Recombinant human Apo-A-1 based HDL mimetic)	Completed	Phase 2 Interventional Single group Open label	>12 Years	HoFH (23)	Effect on Plaque Volume assessed by 3Tesla MRI	https://ClinicalTrials.gov/show/NCT01412034
NCT03510715	Alirocumab	Completed	Phase 3 Interventional Single group Open label	8 - 17 Years	HoFH (18)	Efficacy, Safety, Tolerability	https://ClinicalTrials.gov/show/NCT03510715
NCT03156621 (ODYSSEY HoFH)	Alirocumab	Completed	Phase 3 Interventional Randomized Masking Triple	> 18 Years	HoFH (69)	Efficacy, Safety, Tolerability	https://ClinicalTrials.gov/show/NCT03156621
NCT03403374 (RAMAN)	Evolocumab	Completed	Phase 4 Interventional Single group Open label	12 - 80 Years	HoFH (30)	Efficacy, Safety, Tolerability	https://ClinicalTrials.gov/show/NCT03403374
NCT01624142 (TAUSSIG)	Evolocumab	Completed	Phases 2 & 3 Interventional Single group Open label	12 - 80 Years	Severe FH (300)	Efficacy, Safety, Tolerability	https://ClinicalTrials.gov/show/NCT01624142

NCT02624869 (HAUSER-OLE)	Evolocumab	Completed	Phase 3<br>Interventional<br>Single group<br>Open label	10 - 17 Years	FH (163)	Efficacy, Safety, Tolerability	https://ClinicalTrials.gov/show/NCT02624869
NCT01588496 (TESLA)	Evolocumab	Completed	Phases 2 & 3<br>Interventional<br>Randomized<br>Masking Quadruple	12 - 80 Years	HoFH (58)	Efficacy,	https://ClinicalTrials.gov/show/NCT01588496
NCT04031742	IBI306 (PCSK9 Monoclonal AB)	Active, not recruiting	Phases 2-3<br>Interventional<br>Single group<br>Open label	18 – 80 Years	HoFH (30)	Efficacy, Safety, Tolerability	https://ClinicalTrials.gov/show/NCT04031742
NCT04515927	JS002 (Monoclonal PCSK9 inh)	Active, not recruiting	Phase 2<br>Interventional<br>Single group<br>Open label	12 - 75 Years	Hyperlipemia (30)	Efficacy, Safety, Tolerability	https://ClinicalTrials.gov/show/NCT04515927
NCT04034485	Lerodalcibep vs Evolocumab	Active, not recruiting	Phase 3<br>Interventional<br>Randomized<br>Cross-over<br>Open label	> 10 Years	HoFH (70)	Efficacy, Safety, Tolerability	https://ClinicalTrials.gov/show/NCT04034485
NCT04455581	Recaticimab (Monoclonal PCSK9 inh)	Unknown status	Phase 2<br>Interventional<br>Single group<br>Open label	18- 80 Years	FH (8)	Efficacy, Safety, Tolerability	https://ClinicalTrials.gov/show/NCT04455581
NCT04948008	IBI306 **(**Monoclonal PCSK9 inh)	Recruiting	Phases 2 & 3<br>Interventional<br>Randomized<br>Single group<br>MaskingTriple	18 - 80 Years	HoFH (8)	Efficacy, Safety, Tolerability	https://ClinicalTrials.gov/show/NCT04948008
NCT03933293	Ebronucimab (Monoclonal PCSK9 inh)	Unknown status	Phase 2<br>Interventional<br>Randomized	> 18 Years	HoFH (59)	Efficacy, Safety, Tolerability	https://ClinicalTrials.gov/show/NCT03933293

			Masking Quadruple				
NCT01109368	Standart care	Recruiting	Observational<br>Cohort<br>Prospective	Child, Adult	HoFH (60)	Change in disease progression	https://ClinicalTrials.gov/show/NCT01109368
NCT04681170	Lomitapide	Recruiting	Phase 3<br>Interventional<br>Randomized<br>Open label	5 -17 Years	HoFH (45)	Efficacy, Safety, Tolerability	https://ClinicalTrials.gov/show/NCT04681170
NCT01556906	Lomitapide	Completed	Phase 2<br>Interventional<br>Single group<br>Open label	> 13 Years	HoFH (6)	Safety, Tolerability and Efficacy	https://ClinicalTrials.gov/show/NCT01556906
NCT02173158	Lomitapide	Completed	Phase 3<br>Interventional<br>Single group<br>Open label	> 18 Years	HoFH (9)		https://ClinicalTrials.gov/show/NCT02173158
NCT00943306	Lomitapide	Completed	Phase 3<br>Interventional<br>Single group<br>Open label	> 18 Years	HoFH (19)	Efficacy, Safety, Tolerability<br>Long term	https://ClinicalTrials.gov/show/NCT00943306
NCT00730236	Lomitapide	Completed	Phase 3<br>Interventional<br>Single group<br>Open label	> 18 Years	HoFH (29)		https://ClinicalTrials.gov/show/NCT00730236
NCT02135705 (LOWER Registry)	Lomitapide	Recruiting	Observational<br>ModelCohort|Time<br>PerspectiveProspective	Child, Adult,	HoFH (300)		https://ClinicalTrials.gov/show/NCT02135705
NCT00079846	Implitapide (MTP inh)	Terminated	Phase 2<br>Interventional<br>Randomized<br>Single group<br>Masking Double	8 - 70 Years	FH (60)		https://ClinicalTrials.gov/show/NCT00079846

NCT00607373 (RADICHOL 1)	Mipomersen	Completed	Phase 3<br>Interventional<br>Randomized<br>Masking Quadruple	> 12 Years	HoFH (51)	Efficacy, Safety, Tolerability	https://ClinicalTrials.gov/show/NCT00607373
NCT00477594	Mipomersen	Completed	Phase 2<br>Interventional<br>Randomized<br>Open label	> 12 Years	HoFH (21)	Efficacy, Safety, Tolerability	https://ClinicalTrials.gov/show/NCT00477594
NCT00694109	Mipomersen	Completed	Phase 3<br>Interventional<br>Single group<br>Open label	> 12 Years	HoFH (144)	Long term Efficacy, Safety, Tolerability	https://ClinicalTrials.gov/show/NCT00694109
NCT00280995	Mipomersen	Completed	Phase 2<br>Interventional<br>Randomized<br>Open label	> 18 Years	HoFH (12)	Dose-escalating Safety	https://ClinicalTrials.gov/show/NCT00280995
NCT02651675	AAV directed hLDLR gene therapy	Completed	Phases 1& 2<br>Interventional<br>Single group<br>Open label	> 18 Years	HoFH (9)	Efficacy, Safety, Tolerability	https://ClinicalTrials.gov/show/NCT02651675
NCT03135184	Device HDL Therapeutics PDS-2 System	Unknown status	Interventional<br>Single group<br>Open label	> 12 Years	HoFH (6)	Efficacy, Safety, Tolerability	https://ClinicalTrials.gov/show/NCT03135184
NCT01841684	Anacetrapib (CETP inh)	Terminated	Phase 3<br>Interventional<br>Randomized<br>Masking Double	> 18 Years	HeFH & HoFH (2)	Efficacy, Safety, Tolerability	https://ClinicalTrials.gov/show/NCT01841684
NCT02472535	Seladelpar (MBX-8025) specific PPAR-δ agonist.	Completed	Phase 2<br>Interventional<br>Single group<br>Open label	> 18 Years	HoFH (13)	Efficacy, Safety, Tolerability	https://ClinicalTrials.gov/show/NCT02472535

NCT04080050	RGX-501 Single (IV) (LDLR) Gene Therapy	Active, not recruiting	Observational	> 18 Years	HoFH (8)	Efficacy, Safety, Tolerability	https://ClinicalTrials.gov/show/NCT04080050
NCT03018678	Screening Protocol for a Gene Therapy Trial	Completed	Observational Prospective	> 18 Years	HoFH (21)		https://ClinicalTrials.gov/show/NCT03018678
NCT02722408 (COBALT-1)	Gemcabene	Completed	Phase 2 Interventional Single group Open label	> 17 Years	HoFH (8)	Efficacy, Safety, Tolerability	https://ClinicalTrials.gov/show/NCT02722408
NCT00515307	Bone Marrow Stem Cells (Allogenic Hepatocyte Transplantation)	Completed	Phase 1 Interventional Single group Open label	Child, Adult,	HoFH (1)	Efficacy, Safety, Tolerability	https://ClinicalTrials.gov/show/NCT00515307
NCT00263081	Lapaquistat (squalene synthase inhibitor)	Terminated	Phase 3 Interventional Randomized Masking Quadruple	> 8 Years	HoFH (44)	Efficacy, Safety, Tolerability	https://ClinicalTrials.gov/show/NCT00263081
NCT00705211	Ezetimibe	Completed	Observational Prospective	Child, Adult,	Hypercholesterolemia, FH, Homozygous Sitosterolemia (All:1794)	Efficacy, Safety, Tolerability in Japanese Subjects	https://ClinicalTrials.gov/show/NCT00705211
NCT00704444	Ezetimibe	Completed	Observational Prospective	Child, Adult,	Hypercholesterolemia, FH, Homozygous Sitosterolemia (All 11332)	12-week postmarketing Efficacy, Safety, Tolerability	https://ClinicalTrials.gov/show/NCT00704444
NCT03884452 (MK-0653-018)	Ezetimibe	Completed	Phase 3 Interventional Randomized	> 12 Years	FH (50)	Efficacy, Safety, Tolerability	https://ClinicalTrials.gov/show/NCT03884452

			Masking Double				
NCT00704535	Ezetimibe	Completed	Observational Prospective	> 18 Years	Primary Hypercholesterolemia|HoFH (4105)	Efficacy, Safety, Tolerability in a Select Population of Filipinos	https://ClinicalTrials.gov/show/NCT00704535
NCT01070966	Vytorin (Ezetimibe) Reexamination Study (0653A-174)	Completed	Observational Prospective	> 10 Years	Primary Hypercholesterolemia and HoFH (2089)	Efficacy, Safety, Tolerability	https://ClinicalTrials.gov/show/NCT01070966
NCT03885921 (P01417/MK-0653-019)	Ezetimibe	Completed	Phase 3 Interventional Randomized Masking Quadruple	> 12 Years	HoFH (44)	Efficacy, Safety, Tolerability	https://ClinicalTrials.gov/show/NCT03885921
NCT02434497	Rosuvastatin 20mg	Completed	Phase 3 Interventional Single group Open label	6 - 18 Years	HoFH (9)	Efficacy, Safety, Tolerability	https://ClinicalTrials.gov/show/NCT02434497
NCT02226198 (HYDRA)	Rosuvastatin 20mg	Completed	Phase 3 Interventional Randomized MaskingTriple	6 - 17 Years	HoFH (20)	Efficacy, Safety, Tolerability	https://ClinicalTrials.gov/show/NCT02226198

Abrrevations FH; Familial Hypercholesterolemia, HoFH, Homozygous Familial Hypercholesterolemia; HeFH; Heterozygous Familial Hypercholesterolemia, LDLR; Low density lipoprotein receptor,
